# High prevalence of acetabular rim osteophytes after hip arthroscopy for treatment of FAI

**DOI:** 10.1186/s12891-022-05038-w

**Published:** 2022-01-19

**Authors:** Guanying Gao, Rongge Liu, Hanmei Dong, Yingfang Ao, Jianquan Wang, Yan Xu

**Affiliations:** Institute of Sports Medicine, Beijing Key Laboratory of Sports Injuries, Peking University Third Hospital, 49 North Garden Road, Haidian District, Beijing, 100191 China

**Keywords:** Acetabular rim osteophytes, Femoroacetabular impingement, Hip arthroscopy

## Abstract

**Background:**

Few studies mentioned acetabular rim osteophytes (ARO) after arthroscopy for femoroacetabular impingement (FAI) in follow-up after primary hip arthroscopy. We found that many patients had postoperative ARO, which may lead to recurrent or secondary pincer-type deformity after primary hip arthroscopy for FAI and postoperative ARO sometimes even led to revision surgery. It is necessary to carry out related research on ARO.

**Methods:**

We respectively evaluated consecutive cases who underwent hip arthroscopy in our hospital between January 2008 and January 2020. Radiographic examination was obtained for all patients preoperatively and postoperatively. Another CT scan was performed at least 6 months after surgery at final follow-up. Preoperative patient-reported outcomes (PROs) and PROs at final follow-up were obtained, including visual analog scale (VAS) for pain and modified Harris Hip Score (mHHS). The volume of ARO was calculated using mimics 21.0 software. According to the material of anchors and whether the anchors were used, patients were divided into absorbable group, non-absorbable group and no anchor group.

**Results:**

A total of 71 patients were finally included in this study. Patients with postoperative ARO had higher VAS at final follow-up (P<0.05). Patients without postoperative ARO had higher mean mHHS at final follow-up (*P* = 0.08) and higher percentage of passing minimal clinical important difference. The percentage and volume of postoperative ARO was significantly higher in patients who underwent acetabuloplasty and labral repair (P<0.05). The percentage and volume of postoperative ARO in absorbable group were significantly higher than the other groups (P<0.05).

**Conclusion:**

There is a high percentage of ARO after hip arthroscopy for treatment of FAI and patients who have undergone labral repair and acetabuloplasty are more likely to have postoperative ARO. Using of absorbable anchors may increase the possibility and volume of postoperative ARO. Postoperative ARO may predict a worse clinical outcome.

## Background

Femoroacetabular impingement (FAI) is an important and common cause of hip pain, which is characterized by pathologic contact between the femoral head and acetabulum secondary to bony deformity [1]. FAI causes chondral injury and labral damage and may play an etiologic role in hip osteoarthritis (OA) cases [2, 3]. Over the past decade, hip arthroscopic surgery for treatment of FAI has developed rapidly, becoming a common technique [4]. Recent studies have proved the good clinical outcomes of hip arthroscopy for FAI and a high percentage of patients return to sport activities, with a low rate of complications and reoperation through long-term clinical follow-up [4–8]. However, there are few studies on radiographic follow-up. In our daily work, we found that many patients had acetabular rim osteophytes (ARO), which may lead to recurrent or secondary pincer-type deformity after primary hip arthroscopy for FAI and postoperative ARO sometimes even led to revision surgery. Studies have proved that the main indication for revision is a candidate who has symptoms due to residual cam- or pincer-type deformity [9–11]. However, few studies mentioned ARO after arthroscopy for FAI in follow-up after primary hip arthroscopy or in revision surgery. Superolateral osteophytes of the acetabulum after total hip arthroplasty (THA) has been reported by previous study and the role of these osteophytes remains uncertain [12]. Sebastian et al. [13] found that ARO may result in impingement and limited range of motion (ROM) in flexion, 90° of flexion with internal rotation, and external rotation through computerized virtual surgery. So we hypothesized that postoperative ARO after hip arthroscopy for treatment of FAI may have influence on the clinical outcomes.

The purpose of this study was to evaluate the prevalence and possible influential factors of ARO after hip arthroscopy for treatment of FAI through clinical and CT follow-up.

## Methods

### Patients

We respectively evaluated consecutive cases who underwent hip arthroscopy in our hospital between January 2008 and January 2020. The inclusion criteria were as follows: (1) patients who were diagnosed with FAI and underwent hip arthroscopy for treatment; and (2) patients who had preoperative CT, CT 1 day after surgery and CT performed at least 6 months after surgery at final follow-up. Patients who had subspinal impingement, borderline developmental dysplasia of the hip, ischiofemoral impingement, Legg-Calve-Perthes disease, avascular necrosis, pigmented villonodular synovitis, synovial chondromatosis or OA with Tönnis grade ≥ 2 and patients who could not complete the follow-up were excluded from the study. Patients with prior hip surgery were also excluded. All participants signed informed consent. The study was approved by the Ethics Committee of the Third Hospital of Peking University. All methods were performed in accordance with the guidelines and regulations of the Ethics Committee of the Third Hospital of Peking University.

### Surgical technique

All patients underwent standard supine approach hip joint arthroscopy as described by previous studies [11]. In brief, traction was applied to the operative extremity to ensure that the operative side hip joint space was 8 to 10 mm under fluoroscopic guidance. A detailed inspection of the central compartment was performed to assess the acetabular rim, acetabular labrum, articular cartilage and ligamentum teres. Labral repair was performed according to the nature of injury. Femoral osteoplasty and acetabuloplasty were performed to treat FAI. Material of anchor was also recorded. Capsular closure was routinely done at the end of surgery.

### Clinical and radiographic follow-up

**Fig. 1:**
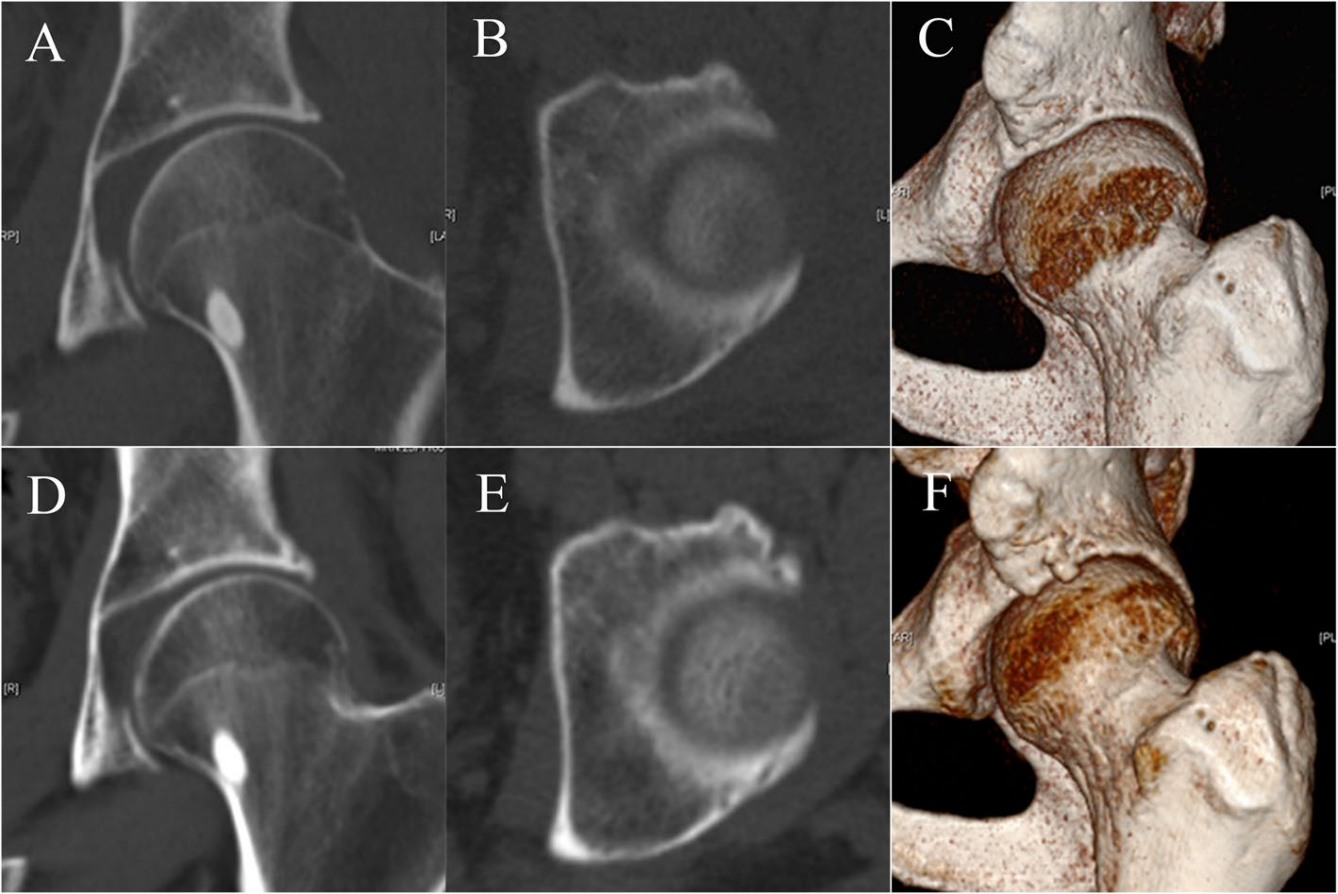
**A**, **D**. Coronal CT 1 day after surgery and at final follow-up. **B**, **E**. Axial CT 1 day after surgery and at final follow-up. **C**, **F**. 3D reconstruction CT 1 day after surgery and at final follow-up

Supine anteroposterior hip radiographs, cross-table lateral radiographs, CT images, and MR images were obtained for all patients preoperatively. Cross-table lateral radiographs and CT images were obtained for all patients 1 day after surgery. Another CT scan was performed at least 6 months after surgery at final follow-up (Fig. 1). Preoperative patient-reported outcomes (PROs) and PROs at final follow-up were obtained, including visual analog scale (VAS) for pain and modified Harris Hip Score (mHHS). Postoperative PROs were recorded at the same time patients underwent CT follow-up. For the mHHS, minimal clinical important difference (MCID) was defined to be 8 by Kemp et al. [14], and the patient acceptable state score (PASS) score was defined to be 74 by Chahal et al. [15].

### Measurement of the volume of ARO

**Fig. 2:**
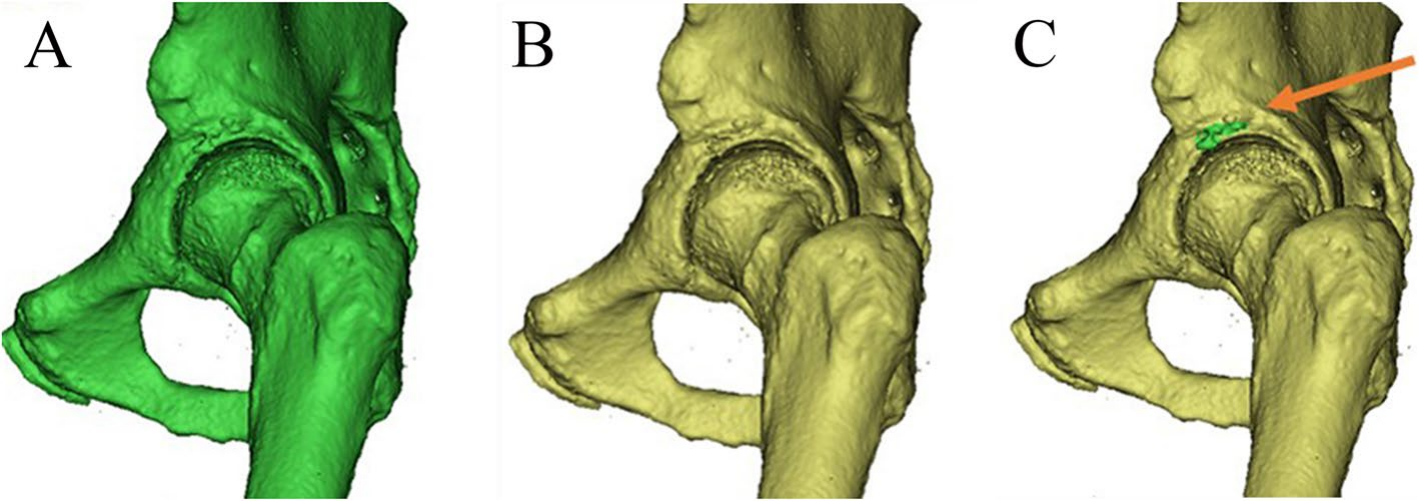
Mimics 21.0 software was used to measure the volume of ARO. Objects with ARO at final follow-up (**A**) and without ARO 1 day after surgery (**B**) were created. **C**. Overlapping objects (green area indicated by the orange arrow) showed the ARO

Mimics 21.0 software was used for the measurement of the volume of ARO (Fig. 2). CT scans (GE Revolution, slice thickness of 0.625 mm, 120 kV, 200 mAs) obtained 1 day after surgery and at final follow-up in DICOM format were used for measurement. Firstly, we chose range of the threshold of Hounsfield Units (HU) values and created a mask that contained ARO (Fig. 2A). A reasonable range of the threshold of HU was used, in which the bony structure was included and artifacts were excluded. Then, the object of hip at final follow-up based on this mask was created in high quality and the volume of this object was measured (Fig. 2A). Then we edited the created mask and used erase tool and draw tool to remove the ARO compared with the postoperative CT scans collected 1 day after surgery. Then the object of hip 1 day after surgery was created and the volume was also recorded (Fig. 2B). The difference between the two objects was considered as the volume of the ARO (Fig. 2C).

### Statistics

Continuous variables with a normal distribution in the baseline data between groups were examined using the independent samples *t*-test. The two-tailed paired *t*-test was used to evaluate significance between preoperative PROs and PROs at final follow-up. Percentages were compared using the Chi-square test. *P* values <.05 were considered statistically significant. All statistical analyses were performed with SPSS Statistics, version 22 (IBM).

## Results

As shown in Table 1, a total of 71 patients (71 hips; 33 male and 38 females; mean age: 36.3 years; age range: 15–65 years) were finally included in this retrospective study. There were 35 left sides and 36 right sides. The mean body mass index (BMI) was 22.2 (range, 16.5–31.2). According to the material of anchors and whether the anchors were used, patients were divided into absorbable group, non-absorbable group and no anchor group. The arthroscopic diagnoses and procedure were shown in Table 2.

**Table 1:** Demography

Parameter	Data
Number of hips	71
Age, y, mean (range)	36.3 (15–65)
Gender	
Male	33 (46.5%)
Female	38 (53.5%)
Side	
Left	35 (49.3%)
Right	36 (50.7%)
BMI, mean (range)	22.2 (16.5–31.2)
Cases with ARO	37 (52.1%)
Cases without ARO	34 (47.9%)
Absorbable group	55 (77.5%)
Non-absorbable group	12 (16.9%)
No anchor group	4 (5.6%)
Mean follow-up time, month (range)	28.1 (6–86)

Unless otherwise specified, data are numbers of patients, with percentages in parentheses

**Table 2:** Arthroscopic diagnoses and procedure

	Number
Diagnosis	
Cam-type FAI	70 (98.6%)
Pincer-type FAI	51 (71.8%)
Labral tear	67 (94.4%)
Arthroscopic procedure	
Labral repair	67 (94.4%)
Femoral osteoplasty	70 (98.6%)
Acetabuloplasty	51 (71.8%)

Data are numbers of patients, with percentages in parentheses

The mean follow-up time after surgery was 28.1 months (range, 6–86 months). The PROs at final follow-up was at the same time of the CT follow-up. As shown in Table 3, mean mHHS was 49.6 ± 13.9 (range, 19–67) and mean VAS was 4.4 ± 1.6 (range, 1–9) before surgery for all patients. At the final post-operative follow-up, mean mHHS was 78.4 ± 10.6 (range, 52–91) and mean VAS was 0.9 ± 1.1 (range, 0–5). At final follow-up, 78.9% of patients surpassed the MCID and 95.8% of patients achieved the PASS. All results demonstrated statistically significant improvement (*P* < 0.05).

**Table 3:** PROs of different groups

	mHHS before surgery	mHHS at final folow-up	VAS before surgery	VAS at final folow-up
All patients	49.6 ± 13.9	78.4 ± 10.6	4.4 ± 1.6	0.9 ± 1.1
Patients with ARO	48.0 ± 13.4	73.6 ± 10.1	4.5 ± 1.7	1.2 ± 1.1
Patients without ARO	50.9 ± 14.3	81.3 ± 11.0	4.3 ± 1.8	0.7 ± 0.7

Values are the mean ± SD

For patients with postoperative ARO, mean mHHS and VAS was 48.0 ± 13.4 (range, 11–75) and 4.5 ± 1.7 (range, 1–9) before surgery and 73.6 ± 10.1 (range, 51–89) and 1.2 ± 1.1 (range, 0–5) at the final post-operative follow-up (Table 3). At final follow-up, 71.8% of patients surpassed the MCID and 95.8% of patients achieved the PASS. For patients without postoperative ARO, mean mHHS and VAS was 50.9 ± 14.3 (range, 15–77) and 4.3 ± 1.8 (range, 0–9) before surgery and 81.3 ± 11.0 (range, 51–91) and 0.7 ± 0.7 (range, 0–5) at the final post-operative follow-up. At final follow-up, 81.7% of patients surpassed the MCID and 93.0% of patients achieved the PASS. There was no significant difference in preoperative mHHS and VAS between patients with and without postoperative ARO. However, patients with postoperative ARO had higher VAS than patients without ARO at final follow-up (P<0.05). Patients with postoperative ARO had lower mean mHHS at final follow-up, but there was no statistical difference (*P* = 0.08).

Among 71 cases, there were 37 (52.1%) cases who had ARO during CT follow-up (Table 1). The mean volume of ARO of 37 cases was 31.9 ± 21.2 mm^3^ (range, 12.1–96.0 mm^3^). In patients who underwent acetabuloplasty, 25 (49.0%) in 51 patients were found postoperative ARO. In patients who underwent labral repair, 32 (47.8%) in 67 patients were found postoperative ARO. In patients who did not underwent acetabuloplasty or labral repair, 1 (25.0%) in 4 patients were found postoperative ARO. The percentage and volume of postoperative ARO was significantly higher in patients who underwent acetabuloplasty and labral repair (P<0.05).

In absorbable group, 32 (58.2%) in 55 cases were found postoperative ARO and the mean volume of ARO was 31.9 ± 21.2 mm^3^ (range, 12.4–96.7 mm^3^) (Table 4). In non-absorbable group, 4 (33.3%) in 12 cases were found postoperative ARO and the mean volume was 18.9 ± 12.9 mm^3^ (range, 11.9–46.3 mm^3^). In no anchor group, 1 (25.0%) in 4 cases were found postoperative ARO and the volume was 19.4 mm^3^. The percentage and volume of postoperative ARO in absorbable group were significantly higher than the other groups (P<0.05).

**Table 4:** Percentage and volume of ARO in different groups

	Percentage of ARO (%)	Volume of ARO (mean ± SD, mm^3^)
All patients	52.1	31.9 ± 21.2
Absorbable group	58.2 ^α, β^	34.6 ± 21.9 ^γ, δ^
Non-absorbable group	33.3 ^α^	18.9 ± 12.9 ^γ^
No anchor group	25.0 ^β^	19.4 ± 0 ^δ^
Patients who underwent labral repair	47.8 ^ε^	30.8 ± 19.4 ^η^
Patients who underwent acetabuloplasty	49.0 ^ζ^	35.2 ± 21.1 ^θ^
Patients who didn’t underwent labral repair or acetabuloplasty	25.0 ^ε, ζ^	19.4 ± 0 ^η, θ^

^α, β, γ, δ, ε, ζ, η, θ^ The same letter indicates significant statistical difference (*P*<0.05)

## Discussion

In this study, we found that a high percentage of ARO after hip arthroscopy for treatment of FAI. Patients who have undergone labral repair and acetabuloplasty are more likely to have postoperative ARO. The percentage and volume of postoperative ARO in absorbable group were significantly higher than non-absorbable and no anchor group. Postoperative ARO may predict a worse clinical outcome.

There were many clinical follow-up studies that proved good outcomes of hip arthroscopy for treatment of FAI [4, 5, 7, 8]. However, there were few researches to study the postoperative changes after femoral osteoplasty, acetabuloplasty, labral repair or labral debridement through radiographic follow-up. Postoperative ARO were found in many patients in our daily work. Residual cam- or pincer-type deformity were regarded as the main reason of revision surgery [9–11, 16–19]. We thought postoperative ARO may lead to recurrent or secondary pincer-type deformity or secondary SSI, which could be a cause of revision surgery. Postoperative ARO was seldom discussed in previous clinical follow-up study or studies on revision hip arthroscopy. Mao et al. [12] reported superolateral osteophytes of the acetabulum after total hip arthroplasty. The role of these osteophytes remains uncertain and the authors thought osteophytes of the acetabulum should play a role in stabilizing the acetabular cup. Sebastian et al. [13] found that ARO may result in impingement in 7 to 8 o’ clock and 1 to 2 o’ clock of the acetabulum through computerized virtual surgery, which may have impact on ROM and should be removed during THA. These studies could inspire us to further study the ARO after hip arthroscopy.

Mean mHHS and VAS of patients with and without postoperative ARO were 73.6 ± 10.1, 1.2 ± 1.1, 81.3 ± 11.0 and 0.7 ± 0.7 at the final post-operative follow-up, respectively. Patients with postoperative ARO had higher VAS than patients without ARO at final follow-up (P<0.05). Patients with postoperative ARO had lower mean mHHS at final follow-up, but there was no statistical difference (*P* = 0.08). Valente et al. [20] evaluated 150 hips in asymptomatic, non-osteoarthritic adult hips and reported ARO was present in 96% of the hips, with an average size of 1.78 mm. So it’s hard to say if these ARO will produce symptoms. However, these new generated ARO in our study were much larger than normal ARO reported by Valente et al. In this study, patients with postoperative ARO had relatively worse mHHS and VAS at final follow-up. Patients without ARO had a higher percentage of passing MCID. So we thought postoperative ARO may predict a worse clinical outcome and ARO should be avoided as much as possible. Deepening the implant depth of absorbable anchors, using of nonsteroidal anti-inflammatory drugs (NSAIDs) and using of radiofrequency to treat cortical bone around the surface of anchors may help reducing the incidence of ARO. Further studies and follow-up are needed to provide evidence on how to reduce the occurrence of ARO.

In this study, 25 (49.0%) in 51 patients who underwent acetabuloplasty were found postoperative ARO. In patients who underwent labral repair, 32 (47.8%) in 67 patients were found postoperative ARO. In patients who did not underwent acetabuloplasty, labral repair or reconstruction, 1 (25.0%) in 4 patients were found postoperative ARO. The percentage and volume of postoperative ARO was significantly higher in patients who underwent acetabuloplasty and labral repair. So surgical operation has an important influence on postoperative ARO. Removing cortical bone to freshen the acetabulum rim may stimulate acetabular hyperplasia and produce osteophytes.

Thirty-two (58.2%) in 55 cases who used absorbable anchors were found postoperative ARO and the percentage and volume of postoperative ARO in absorbable group were significantly higher than the other groups (P<0.05). The material of anchors has an important influence on postoperative ARO. Absorbable materials may cause bone stimulation and generate ARO in the process of absorption. Further research is needed to clarify this phenomenon and mechanism.

### Limitations

This study has some potential limitations. Firstly, the number of patients in no anchor group was relatively small because most patients had labral tear and need anchor for suture. Secondly, PROs only included mHHS and VAS.

## Conclusion

There is a high prevalence of ARO after hip arthroscopy for treatment of FAI and patients who have undergone labral repair and acetabuloplasty are more likely to have postoperative ARO. Using of absorbable anchors may increase the possibility and volume of postoperative ARO. Postoperative ARO may predict a worse clinical outcome.

## Data Availability

All relevant data supporting the conclusions are included within the article and tables. The datasets used and/or analysed during the current study available from the corresponding author on reasonable request.
